# The generative mechanism of workplace boomerang behavior: a grounded theory approach

**DOI:** 10.3389/fpsyg.2026.1755787

**Published:** 2026-04-09

**Authors:** Zehui Tian, Qinghong Yuan, Xiaohong Dong, Chen Shen

**Affiliations:** 1 School of Management, Hebei Geo University, Shijiazhuang, China; 2 School of Business, Nankai University, Tianjin, China

**Keywords:** boomerang behavior, ex-employees, grounded theory, previous organization, turnover

## Abstract

**Background:**

As employee turnover intensifies and the talent shortage grows, boomerang employment—the phenomenon of former employees returning to their previous organizations—has become increasingly common. However, theoretical understanding of how this behavior occurs remains limited.

**Objective:**

This study aims to explore the formation mechanisms of workplace boomerang behavior and construct a theoretical model to explain this process.

**Methods:**

Employing a grounded theory approach, we collected data from two sources: online secondary materials and semi-structured interviews with former employees who had returned to their previous companies. The data were systematically analyzed through open coding, axial coding, and selective coding to identify core categories and their interrelationships.

**Results:**

Anchoring factors (rational, emotional, matching, normative) and driving factors (career, contextual, cognitive) shape boomerang intention (cognitive commitment and behavioral enactment), ultimately leading to boomerang behavior. Organizational opportunities (talent gaps, value assessments, industry ecology) are critical for enabling boomerang behavior.

**Conclusion:**

Our work makes the following theoretical advancements. First, by systematically examining how boomerang behavior emerges, this study shifts the analytical focus from “outcome analysis” to “process genesis.” Second, by investigating the critical role of the former organization in the formation of boomerang behavior, this study builds a “bilateral interaction” decision model. Third, by framing boomerang behavior as a distinct form of situated job choice, this study addresses the explanatory shortcomings of traditional job-choice theories in this context.

## 1 Introduction

Against the backdrop of increasing workforce mobility and the recognition that former employees often possess critical knowledge and resources (Makarius et al., 2025a; Fulmer et al., 2024), organizations are becoming increasingly aware of the advantages of rehiring former employees. These advantages encompass the introduction of new expertise, access to valuable social capital, mitigation of hiring risks, and reduced onboarding and socialization costs (Wang and Cotton, 2025; Kehoe et al., 2024; Bidwell and Keller, 2014; Snyder et al., 2021; Raveendra and Satish, 2021). Consequently, a growing number of firms are adopting open attitudes toward workplace boomerang behavior. For instance, Alibaba Group has rehired over 2,000 former employees, Air New Zealand recalled 1,000 former employees in 2022, and Ping An Insurance launched the “Returning Birds” program to encourage former employees to return. Statistics indicate that boomerang hires now account for 10–20% of total recruitment, with this figure steadily rising (Raveendra and Satish, 2021; Keller et al., 2020; Swider et al., 2017;). Arnold et al. (2020) further highlight that former employees have emerged as a third critical talent source, complementing external hiring and internal promotions.

Hom et al. (2017) have called for greater scholarly attention to the phenomenon of workplace boomerang behavior. However, extant research is marked by three interrelated gaps that limit our understanding of its generative logic. First, a skewed focus on “consequences” over “origins.” Existing research has predominantly focused on its consequences, such as returnees’ attitudes, behaviors, and performance (Grohsjean et al., 2025; Snyder et al., 2021; Keller et al., 2020; Swider et al., 2017; Arnold et al., 2020; Maier et al., 2021), while the generative mechanisms—why and how former employees return—remain underexplored (Sullivan and Al Ariss, 2021). Second, an oversimplified “unilateral” rather than “bilateral” framework. Since boomerang behavior results from bilateral decision-making between former employees and organizations, a dual-perspective approach is essential to comprehensively understand its emergence (Dlouhy et al., 2025). Third, a theoretical disconnect from its distinct nature as “job choice.” Boomerang behavior fundamentally represents a kind of job choice. Although traditional job choice theories—such as expectancy theory (Maestas et al., 2023; Wanous et al., 1983), person-organization fit models (Tanwar and Kumar, 2019; Chapman et al., 2005), and social influence theory (Rocha and Praag, 2020)—provide partial explanations, they fail to fully account for the unique dynamics of boomerang behavior, such as the stigma associated with perceived disloyalty, pre-existing emotional ties with the former employer, and reduced information asymmetry compared to external hires.

To systematically address these three gaps, this study employs a grounded theory approach to investigate the generative mechanisms of workplace boomerang behavior. Our work makes the following theoretical advancements. First, by systematically examining how boomerang behavior emerges, this study shifts the analytical focus from “outcome analysis” to “process genesis.” Second, by investigating the critical role of the former organization in the formation of boomerang behavior, this study builds a “bilateral interaction” decision model. Third, by framing boomerang behavior as a distinct form of situated job choice, this study addresses the explanatory shortcomings of traditional job-choice theories in this context.

## 2 Literature review

### 2.1 Job choice and boomerang behavior

Job choice behavior refers to the decision-making process through which individuals evaluate and select employment opportunities (Chapman et al., 2005; Gatewood et al., 1993). Workplace boomerang behavior represents a distinct yet understudied form of this broader behavior (Dlouhy et al., 2025). While traditional job choice research has focused on first-time applicants (e.g., new graduates; Chapman et al., 2005) or general labor market participants (Gatewood et al., 1993), the unique dynamics of boomerang employees—who re-evaluate former employers— is an emerging trend (Sullivan and Al Ariss, 2021).

Three dominant theoretical lenses explain job choice: Expectancy Theory posits that job seekers act as rational utility maximizers, weighing attributes like compensation and work conditions (Maestas et al., 2023; Wanous et al., 1983). Person-Environment Fit Model emphasizes compatibility between individual values and oganizational culture (Tanwar and Kumar, 2019; Chapman et al., 2005). Social Influence Theory hrighlights the role of peer networks and cultural norms in shaping decisions (Rocha and Praag, 2020). While the aforementioned theories provide foundational explanations for general job choice, they fall short in capturing the dual forces unique to boomerang employment. This theoretical gap is evident in two dimensions that extend beyond conventional explanations: First, the unexplained ‘Pull’ of affective and relational residue. Existing models, while able to account for pulls like compensation or fit, cannot fully incorporate the affective and relational capital that persists after departure—such as nostalgia for the organizational culture or enduring social bonds with former colleagues (Snyder et al., 2021; Schärrer and Sender, 2023). This historical-emotional residue constitutes a distinct pull absent in standard job choice scenarios. Second, the overlooked ‘Push’ from external disillusionment. Similarly, these frameworks overlook the contextual push factor wherein former employees realize that external opportunities are less favorable than anticipated—a recognition that “The grass is always greener on the other side (Raveendra and Satish, 2021).” This disillusionment actively motivates reconsidering a return, a dynamic not addressed by current paradigms. Together, the untheorized relational-affective pull and the disillusionment-driven push form a critical blind spot, underscoring the need for a more integrated framework to fully explain boomerang behavior.

### 2.2 Workplace boomerang behavior

In broad terms, workplace boomerang behavior refers to the phenomenon where former employees return to work for a previous employer after a period of separation (Swider et al., 2017; Arnold et al., 2020; Shipp et al., 2014). This definition captures the essential feature of two distinct employment periods separated by time away from the organization (Makarius et al., 2025b). Shipp et al. (2014) distinguish between two types of boomerangs: planned and unplanned. Planned boomerangs involve predetermined rehiring arrangements, such as seasonal work patterns or temporary leaves for personal reasons, which typically require less complex decision-making processes (Magrizos et al., 2023; Snyder et al., 2021). Unplanned boomerangs occur when employees return without prior intention, often after discovering more favorable conditions at their former workplace (Shipp et al., 2014). This study focuses on the more prevalent and complex unplanned boomerangs, which involve intricate bilateral decision-making between former employees and organizations.

Current research on workplace boomerang behavior has predominantly examined its consequences. For instance, Grohsjean et al. (2025) find that boomerangs, compared to new hires, exhibit more performance assistance toward incumbent former and incumbent new colleagues. Swider et al. (2017) adopted a career learning perspective to demonstrate how knowledge accumulation and interruption factors predict performance among returning basketball players. Keller et al. (2020) drew on knowledge-based theory to compare 2,053 boomerang employees with 10,858 new hires in a healthcare organization, finding that returnees exhibited superior initial performance, particularly in roles requiring internal coordination. Arnold et al. (2020) found that returning employees in a retail chain underperformed compared to both internal promotions and external hires. While Shipp et al. (2014) examined how initial departure reasons and tenure length influence return behavior, Tian et al. (2022) proposed the concept of legacy identification in explaining return intentions, and Dlouhy et al. (2025) offered a comprehensive conceptual model of the boomerang mobility process, the complete generative mechanisms underlying boomerang behavior remain insufficiently explored.

Moreover, as boomerang behavior results from bilateral decision-making between former employees and organizations, understanding the genesis of this mechanism hinges on recognizing that “boomerang” behavior is far from being determined solely by the former employee’s unilateral intention. Classical organizational behavior theories, such as Schneider (1987) Attraction-Selection-Attrition (ASA) framework, have long posited that organizations continually shape themselves through their personnel decisions. Extending this logic to the rehiring context, it is important to note that organizations are not universally receptive to rehiring former employees. The specific conditions under which organizations are more willing to welcome returnees remain an unresolved question. Therefore, to comprehensively understand the emergence of boomerang behavior, a research approach that integrates both individual and organizational perspectives is essential. This necessitates not only examining employees’ return intentions but also systematically exploring the organizational side of the equation—specifically, the conditions under which former employees are (re)defined as viable and valuable candidates.

## 3 Materials and methods

### 3.1 Research methodology

The generative mechanism of workplace boomerang behavior remains an underexplored area in organizational research (Dlouhy et al., 2025), characterized by complex interdependencies among multiple factors. Given the intricate and multifaceted nature of this phenomenon, a grounded theory approach is particularly suitable, as it facilitates theory development through systematic data analysis and inductive reasoning (Corbin and Strauss, 2015; Li et al., 2023). Among the three primary variants of grounded theory—classical, constructivist, and procedural—we adopt the procedural approach, which has been extensively validated in Chinese academic research. Following Corbin and Strauss (2015) structured methodology, we employ a three-stage coding process (open, axial, and selective coding) to systematically analyze the data and develop a coherent theoretical framework.

### 3.2 Data collection

The Internet serves as a valuable source for collecting secondary data in the era of big data (Bischof and Freybe, 2022; Cheong et al., 2023). Given the anonymity, immediacy, and openness of online forums, the abundance of free and authentic discourse available on these platforms meets the fundamental requirements of grounded theory. This study utilizes the Zhihu forum as the data source, employing keywords such as “rehiring,” “boomerang employee,” “former employer,” and “reinstatement” to retrieve 2,220 text entries via web crawler software. The following screening criteria were applied: first, only concrete cases were included to exclude evaluative or promotional content; second, cases were limited to first-person narratives or accounts relayed by colleagues, acquaintances, or relatives to ensure textual reliability; third, planned return events such as seasonal re-employment or post-maternity leave returns were excluded. A total of 304 text entries, comprising 163,477 words, were retained for analysis. In-depth interviews were employed to compensate for the limitations of secondary online data, such as the lack of contextual background information and opportunities for probing questions. The triangulation of online archival data and interviews significantly enhances the robustness of the findings. Interviews conducted via Tencent Meeting proved efficient and cost-effective, while the familiarity of the virtual setting facilitated natural participant responses.

Guided by the principles of theoretical sampling (Glaser and Strauss, 2017; Coyne, 1997), we dynamically selected and interviewed 10 professionals with boomerang employment experiences (from January to March 2024) based on concepts emerging from data analysis, continuing the process until theoretical saturation was reached. The participants (mean age = 37 years) represented diverse sectors, including education, manufacturing, catering, IT, and real estate (see Table 1). The semi-structured interviews employed retrospective questioning to trace the generative mechanisms of boomerang behavior, include questions such as “Could you describe your boomerang employment experience in detail?”, “What motivated your decision to return to your former employer?”, “How did the organization respond to your return?” Each interview lasted 30–60 min and was audio-recorded with consent, yielding 65,546 Chinese characters of transcribed data.

**Table 1:** Interviewees’ demographic profile.

ID	Gender	Age	Education	Industry	Prior tenure (years)	Company type	Interview duration (min)
1	Female	52	Vocational	Education	2	State-owned enterprise	55
2	Female	32	Bachelor	Catering	5	Foreign-invested enterprise	36
3	Male	36	Associate	Construction	7	Domestic private enterprise	30
4	Female	35	Master	Manufacturing	3	Foreign-invested enterprise	49
5	Male	39	Bachelor	Manufacturing	3	Foreign-invested enterprise	46
6	Male	32	Bachelor	Real estate	1	Domestic private enterprise	33
7	Male	42	Bachelor	Insurance	6	Domestic private enterprise	41
8	Female	43	Bachelor	Construction	3	State-owned enterprise	48
9	Male	33	Master	IT	4	Domestic private enterprise	45
10	Female	26	Bachelor	Real estate	1	Domestic private enterprise	53

### 3.3 Data analysis

#### 3.3.1 Open coding

Open coding refers to the process of gradually conceptualizing and categorizing textual data through continuous comparison of content (Corbin and Strauss, 2015). This study adopted word-by-word coding to analyze 284 randomly selected cases from the 304-case text dataset. The process involved: first, labeling the original text; second, grouping initial labels with similar meanings into codes and further refining them into concepts; and finally, abstracting concepts into categories. To ensure coding rigor, three HRM PhD candidates were invited to conduct independent back-to-back coding, followed by repeated comparisons and discussions of the coding results. If coding disagreements arose, the researchers resolved them through a consensus mechanism, engaging in in-depth discussions on specific points of divergence until a unanimous agreement was reached (Laestadius et al., 2024). Ultimately, 1,291 labels, 153 initial codes, 47 concepts, and 13 categories were identified (see Table 2).

**Table 2:** Examples of open coding.

Partial raw data	Codes	Concepts	Categories
“After negotiations, the annual salary increased by 50%”	A1 Salary increase	C1 Salary	Rational anchoring
…	…	…	
“There was a lot of friction before, but after leaving, I missed the people and things at Huawei. I have a Huawei alumni group chat, and during one discussion, I found many shared this nostalgia.”	A21 Nostalgia	C5 Lingering attachment	Emotional anchoring
…	…	…	
“The boss said former employees are always welcome back and would not be treated differently.”	A32 Leader’s welcoming attitude without discrimination	C8 Leader norms	Normative anchoring
…	…	…	
“The job content was almost identical to my previous company, so I could transition seamlessly.”	A59 Job content consistency	C13 Job matching	Matching anchoring
…	…	…	
“I thought I could find a job immediately after quitting, but reality struck the next day. Two companies I had eyed earlier said they had already hired, while another I wasn’t keen on rejected me. One interview felt like they wanted to manipulate me.”	A95 Unable to find suitable job	C26 Career gap	Career-driven
…	…	…	
“Car and mortgage loans”	A115 Debt pressure	C29 Financial pressure	Non-work-specific-driven
…	…	…	
“I had no idea what I liked or wanted to do.”	A119 Lost career direction	C31 Confusion	Cognitive-driven
…	…	…	
“My first thought upon arrival was: I cannot stay here—I need to go back.”	A122 Desire to return	C33 Return expectation	Cognitive commitment
…	…	…	
“I reluctantly called my former boss to ask if I could return.”	A128 Contacted former leader	C35 Proactive outreach	Behavioral enactment
…	…	…	
“The company started a new project and was short-staffed.”	A136 New project staffing shortage	C38 Growth-driven gap	Talent gap
…	…	…	
“My supervisor was highly skilled and a great manager. Rumor had it he previously led a team at a major tech firm and joined our company for equity. Soon, our department became the top performer.”	A144 Strong professional competence	C41 Work capability	Value Assessment
…	…	…	
“There are only four top-tier global HR consulting firms: M, AH, WTW, and HG. The industry is small, and not all are hiring. After three job hops, returning is almost inevitable.”	A153 Small industry circle	C44 Industry capacity	Industry ecology
…	…	…	
“After a week off, I returned to my former employer right after the New Year holiday.”	A155 Return behavior	C46 Return behavior	Boomerang behavior
1,291	153	47	13

#### 3.3.2 Axial coding

Axial coding reorganizes the categories derived from open coding to uncover latent logical relationships among them (Corbin and Strauss, 2015). Through analyzing the correlations among the 13 categories, five main categories were identified: Anchoring Factors, Driving Factors, Organizational Opportunity, Boomerang Intention, and Boomerang Behavior. The relationships between main categories and subcategories, along with their definitions, are presented in Table 3.

**Table 3:** Results of axial coding.

No.	Main categories	Subcategories	Definitions
1	Anchoring factors	Rational anchoring	Former employees’ rational considerations for returning, such as salary and career development.
		Emotional anchoring	Positive, attachment-based emotions tied to the former employer (e.g., nostalgia, gratitude).
		Normative anchoring	Perceived organizational norms toward returnees, including inclusive culture and unbiased attitudes.
		Matching anchoring	Multidimensional fit between the employee and the organization (e.g., person-organization, person-job, person-leader fit).
2	Driving factors	Career-driven	Push factors from career-related setbacks (e.g., unfavorable job market, external role misfit).
		Non-work-specific-driven	Pressures or demands originating from one’s personal life circumstances (e.g., financial strain, family needs)
		Cognitive-driven	Negative psychological triggers arising from an employee’s current situation or new environment (e.g., anxiety, confusion).
3	Organizational opportunity	Talent gap	Organizational staffing needs (e.g., skill shortages, project demands).
		Value assessment	The perceived value of the former employee (e.g., competence, strategic importance).
		Industry ecology	Industry-specific dynamics (e.g., limited competitors, high rehire rates).
4	Boomerang intention	Cognitive commitment	The former employee’s overall attitude toward returning (e.g., desire, regret over leaving).
		Behavioral enactment	Actions taken to facilitate return (e.g., reapplying, negotiating terms).
5	Boomerang behavior	Return behavior	The act of rejoining the former employer.

#### 3.3.3 Selective coding

**Figure 1:**
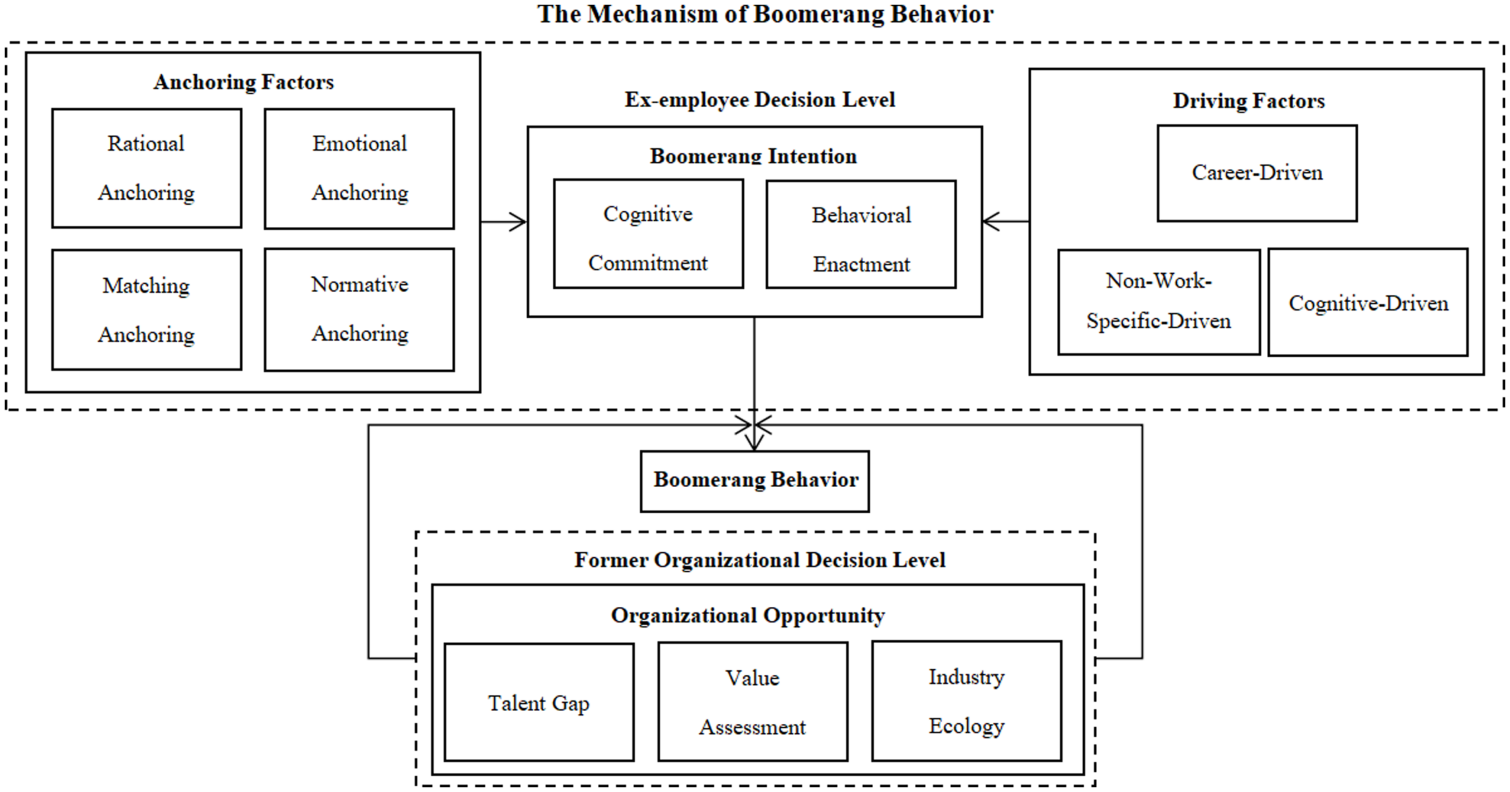
The generative mechanism of boomerang behavior.

Selective coding involves identifying a core category that integrates and abstracts all major categories, thereby constructing a theoretical framework through a coherent “storyline” (Corbin and Strauss, 2015). Based on in-depth analysis of the main categories, this study identifies the generative mechanism of workplace boomerang behavior as the core category. The theoretical model is illustrated in Figure 1. First, the antecedents of boomerang intention and behavior include anchoring factors and driving factors. Anchoring factors comprise rational anchoring, emotional anchoring, matching anchoring, and normative anchoring; driving factors include career-driven, situational-driven, and cognitive-driven motivations. These two sets of factors jointly contribute to former employees’ boomerang intention and behavior. Second, boomerang intention consists of both Cognitive commitment and behavioral enactment, serving as the mediating mechanism between antecedents and the actual boomerang behavior. Third, organizational opportunities—including talent gaps, value assessment, and industry ecology—serve as enabling conditions. When talent shortages exist, the former employee is perceived as valuable, and the industry ecology is closely networked, the likelihood of boomerang behavior is significantly increased.

#### 3.3.4 Theoretical saturation test

Theoretical saturation occurs when no new categories or relationships emerge from additional data (Corbin and Strauss, 2015). To test saturation, the reserved 20 online texts and 10 interview transcripts were coded using the same method. No new categories or relationships were identified, confirming model saturation.

## 4 Results

### 4.1 Anchoring factors and their influence on boomerang intentions/behavior

Anchoring factors refer to the pull forces exerted by the previous organization on boomerang employees (Brazier et al., 2024; Mullet et al., 2000). Among these, rational anchoring is grounded in “efficiency logic,” where the primary objective is to maximize gains. According to expectancy theory (Wanous et al., 1983), job seekers aim to optimize the expected utility of their choices. Barnard (1938) posited that self-interest motives dominate individuals’ decisions to join an organization, suggesting that firms should either enhance incentives (e.g., higher wages) or mitigate disincentives (e.g., reduced working hours). Maier et al. (2021) further demonstrated that core job characteristics often improve upon reemployment.

As social beings, individuals also weigh the perceptions of others (e.g., supervisors, colleagues) in their decision-making (Fishbein and Ajzen, 1975). Normative anchoring, based on “social legitimacy logic”, reflects the social support former employees anticipate when returning (Magrizos et al., 2023; Dlouhy et al., 2025). Notably, given the stigmatization of ex-employees as “disloyal”, discrimination from the previous organization or its members (e.g., peers, leaders) may hinder boomerang intentions (Rocha and Praag, 2020), thereby validating and enriching the role of social influence in career choices.

Matching Anchoring refers to the alignment that former employees perceive between themselves and their former organization, job, supervisor, colleagues, and other relevant entities. Grounded in the person–environment fit theory, the compatibility between an individual and their environment serves as a critical motivator in career decision-making (Tanwar and Kumar, 2019). Given that former employees possess more accurate and profound insights into the fit with their previous organizational context, job role, leadership, and other elements, perceived fit operates as a key driver in their decision to return to the former employer (Maier et al., 2021)—distinguishing it from general career choice scenarios. Dlouhy et al. (2025) pointed out that familiarity—defined as the degree to which a person recognizes or is acquainted with Organization A after the boomerang transition through previous exposure or experience—helps facilitate employees’ return.

Affective anchoring stems from emotional ties between former employees and the previous organization or its constituents (Breitsohl and Ruhle, 2016). In typical job transitions, such preexisting bonds are absent; however, in boomerang scenarios, affective factors—such as nostalgia (Ybema, 2004) or organizational identification (Bardon et al., 2014; Eury et al., 2018)—play a significant role in shaping boomerang intentions and behavior. In summary, anchoring factors (rational, normative, matching, and affective) strengthen former employees’ boomerang intention and behavior.

For example:

“The director even mentioned that if I rejoined, I’d receive back commissions for clients I’d previously secured (anchoring factor). They tried to tempt me with money—I admit, for a split second, that payout made me consider going back (boomerang intention).”

Based on the above, the following proposition is put forward:

*Proposition 1*: Anchoring factors (rational anchoring, normative anchoring, matching anchoring, and affective anchoring) positively influence the formation of boomerang intention and boomerang behavior.

### 4.2 Driving factors and their influence on boomerang intentions/behaviors

Driving factors refer to the difficulties faced by former employees in their current situations (Brazier et al., 2024; Mullet et al., 2000). Career-driven factors involve professional challenges or failures encountered after leaving the previous organization, such as setbacks in new jobs or a harsh job market (Maier et al., 2021). Non-work-specific-driven factors refer to personal life challenges and pressures (Maier et al., 2021), including financial or family-related stress. Cognitive-driven factors reflect psychological struggles experienced after departure, such as anxiety and confusion.

These driving factors tend to increase the boomerang intention and behavior. From one hand, research on memory reconstruction and psychological defense mechanisms suggests that individuals in distress are inclined to recall and amplify past experiences that were emotionally rewarding or brought a sense of achievement, in order to protect themselves from psychological pain, stress, or anxiety. Consequently, current difficulties can enhance positive cognition and emotional attachment to the former organization, thereby increasing the intention and actual behavior of returning (Lee and Sturm, 2017). From other hand, opportunity cost theory offers a rational economic perspective to understand this relationship (Trinks and Scholtens, 2018). For former employees in distress, the cost of giving up alternative opportunities becomes relatively low (Dlouhy et al., 2025), making boomerang intentions and behavior more likely.

In summary, driving factors contribute to stronger return intentions and behaviors. For example:

“After a few months, I wanted to explore the world and decided to resign and join a construction project at Xiaolangdi on the Yellow River, still as a translator. I left with a spirit of adventure, but upon arrival, I felt utterly disheartened. The conditions at the site were extremely poor—it was the middle of winter and freezing cold (Driving Factor). My immediate reaction was: ‘I can’t stay here, I need to go back’ (Return Intention). I immediately called a former supervisor at my previous company (Return Intention).”

Based on the above, the following proposition is put forward:

*Proposition 2*: Driving factors (Career-driven, Non-work-specific-driven, Cognitive-driven) positively influence the formation of boomerang intention and boomerang behavior.

### 4.3 Boomerang intention and its mediating role

Intention refers to the degree to which an individual is willing to attempt a particular behavior and exert effort toward it (Ajzen, 1991). Traditionally, intention is regarded as a psychological state closely linked to behavior. However, other researchers argue that intention itself involves behavior; once an individual intends to act, they are in fact already engaging in goal-directed behaviors (Conradie, 2014; Bratman, 1987). Similarly, the concept of implementation intentions (Gollwitzer, 1999) — whereby individuals form specific “if-then” plans to automate action initiation — illustrates how the intentional process itself can encompass the active planning of future behavior, further substantiating the view that intention involves behavior-oriented components. Empirical evidence supports this perspective. For example, Chapman et al. (2005) found that job search intention includes behaviors such as submitting applications and participating in interviews—behaviors that express intention but precede a final employment decision. This study identifies two dimensions of return intention: cognitive commitment and behavioral enactment. Cognitive commitment refers to the former employee’s overall attitude toward returning to their previous employer, including their expectations about rejoining and any regret over leaving. Behavioral enactment refers to specific actions taken to facilitate a return, such as submitting a job application.

According to the theory of planned behavior, intention plays a critical role in driving actual behavior—the stronger the intention, the higher the likelihood of behavior (Ajzen, 1991; Armitage and Conner, 2001). In the context of boomerang employment, information derived from anchoring and driving factors can influence a former employee’s cognitive intention and degree of effort, ultimately contributing to actual boomerang behavior. For instance:

“He realized that the pressure at his current job was mounting—not only did he have to manage complex financial issues, but he also had to deal with relentless demands from creditors, and at times even had to use his own money to cover company losses (Driving Factor). He began to question whether he had made the wrong decision by giving up a stable job for such a high-risk venture (Boomerang Intention)… Eventually, he quit the startup and returned to his former company (Boomerang Behavior).”

Based on the above, the following proposition is put forward:

*Proposition 3*: Boomerang intention (cognitive commitment and behavioral enactment) positively influence the formation of the boomerang behavior.

### 4.4 Organizational opportunity and its moderating role

According to the Theory of Planned Behavior, the attainability of an opportunity determines the likelihood of behavior occurring. Any behavior requires the existence of objective opportunities and conditions to be realized (Ajzen, 1991). Return-to-work is a bilateral decision-making process between the former employee and the previous organization. Therefore, whether the organization offers reemployment opportunities is a necessary condition for boomerang behavior to occur (Rivera, 2020; Kuhn and Maleki, 2017). This study identifies three forms of organizational opportunity: talent gap, value assessment, and industry ecology. Talent gap refers to the organization’s objective need for human resources. Boomerang behavior is more likely to occur when there is a clear demand for talent. Value assessment involves the extent to which former employees can provide value to the organization. When former employees are seen as valuable assets, the organization is more likely to offer them reemployment opportunities. Industry ecology refers to the overall capacity and interconnectivity within an industry, including market size and information transparency. In industries that are small in scale and closely interconnected, former employees and previous organizations tend to maintain stronger ties, making boomerang behaviors more likely. For example:

“Around 2014, a department head left our company to take a managerial position at a competitor. We made significant efforts to find a replacement—it took over two months and remained unresolved. Despite numerous interviews, there was always something unsatisfactory about the candidates (Organizational Opportunity). Then one day, my supervisor told me that he had already spoken with his former boss—he wanted to come back (Return Intention)… A couple days later, he did return (Return Behavior).”

*Proposition 4*: Organizational opportunity (talent gap, value assessment, and industry ecology) positively moderates the relationship between boomerang intention and behavior, such that the intention is more likely to translate into actual behavior when organizational opportunity is present.

## 5 Discussion

### 5.1 Research findings

Using grounded theory methodology, this study analyzed secondary data and in-depth interview transcripts to construct a theoretical model explaining the mechanisms underlying boomerang behavior. The findings are as follows: First, the study identifies a set of systematic antecedents of boomerang behavior. Anchoring factors include rational anchoring, emotional anchoring, matching anchoring, and normative anchoring. Driving factors include career-driven, non-work-specific-driven, and cognitive-driven challenges. Second, the study finds that boomerang intention comprises two dimensions: Cognitive commitment and behavioral enactment. Anchoring and driving factors influence boomerang behavior through their impact on return intention. Third, the study highlights organizational opportunities that facilitate boomerang behaviors, including talent shortages, value assessments, and industry ecology. These opportunities are essential conditions for the actual occurrence of boomerang behavior.

### 5.2 Theoretical contributions

First, this study addresses a pivotal gap in the literature by shifting the focus from the consequences to the generative mechanisms of boomerang behavior. Prior research has primarily concentrated on the outcomes of boomerang behavior, such as the attitudes, behaviors, and performance of boomerangs (Snyder et al., 2021; Keller et al., 2020; Swider et al., 2017; Arnold et al., 2020; Maier et al., 2021). In contrast, limited attention has been paid to how boomerang behaviors emerge. By identifying the systematic antecedents of boomerang behavior, this study develops an explanatory dynamic process model that addresses this gap. In the absence of this research, the academic discourse would remain focused on describing the outcomes of the boomerang phenomenon, lacking a mechanistic explanation of its formation process.

Second, this study challenges the unilateral view of boomerang employee decision-making in favor of a bilateral perspective that incorporates both the former employee and the previous organization. Since employment decisions are inherently interactive processes involving both parties, the role of the previous organization is critical. However, empirical focus remains predominantly on individual-level factors, such as legacy identification (e.g., Tian et al., 2022). A systematic investigation into the organizational side of the decision is notably absent. This study finds that when talent gaps exist, when former employees are assessed as valuable, and when the industry ecology is tight-knit, organizations are more inclined to offer boomerang opportunities—ultimately enabling boomerang behavior. Thus, by introducing and validating key organizational decision-making factors, this study completes the theoretical picture of boomerang employment, framing it not as a solo act but as a mutually enabled reunion.

Finally, By framing boomerang behavior as a distinctive form of job choice, this study delineates a distinct domain within the broader literature on job choice. Most existing studies focus on job seekers in the open labor market, such as new graduates or job-switchers (Purohit et al., 2021). In contrast, this study examines boomerangs as a unique job choice context, thereby enriching the research landscape of job choice. Furthermore, although traditional studies on job choice have identified a wide range of influencing factors (e.g., compensation and benefits), they often overlook critical elements salient to boomerangs, such as perceived stigma from former colleagues, or deep emotional ties with the previous organization. By extending the context of job choice to the decision-making process of re-entering a former organization, and by elucidating the distinct mechanisms of career choice within this setting, this study offers novel theoretical insights to the job choice literature.

### 5.3 Practical implications

First, organizations should enhance their anchoring factors to increase the likelihood of boomerang behaviors. Among them, rational anchoring aims to maximize gains. To improve rational anchoring, companies should offer competitive compensation, clear career development paths, reasonable workload arrangements, and an optimized institutional environment. Emotional anchoring refers to the emotional attachment to the original employer. To strengthen emotional anchoring, firms should proactively maintain emotional bonds with former employees through full-cycle relationship management, including before, during, and after their departure (Fulmer et al., 2024). Specifically, prior to departure, positive employee-organization relationships should be cultivated to lay the foundation for emotional connection. During the resignation process, organizations should adopt a “parting on good terms” mindset, providing appropriate support and retention efforts. After departure, companies can maintain relationships by sending updates, holiday greetings, commemorative gifts, or organizing reunion events (Dachner and Makarius, 2022). Normative anchoring relates to the perceived organizational norms toward returnees. To enhance normative anchoring, organizations should eliminate biases against boomerang employees, ensure fairness in resource and task allocation, and foster an inclusive organizational culture. Matching anchoring involves the multidimensional fit between the employee and the organization. To improve matching anchoring, companies need to understand the individual strengths and characteristics of former employees to provide well-matched roles, supervisors, and colleagues.

Second, since driving factors (career-driven, non-work-specific-driven, and cognitive-driven) are another key force behind boomerang behavior, organizations should pay close attention to these factors. Career-Driven are push factors from career-related setbacks, non-work-specific-driven are the pressures or demands originating from one’s personal life circumstances, cognitive-driven are negative psychological triggers arising from an employee’s current situation or new environment (e.g., anxiety, confusion). Therefore, enterprises should precisely focus on former employees’ current career status, life pressures, and cognitive-emotional conditions. Specifically, HR and talent acquisition functions should establish an “Alumni Pulse” monitoring protocol to systematically track the career trajectories of high-value alumni through professional networks (e.g., LinkedIn; Cho and Lam, 2021) and structured check-ins, with the goal of identifying signals of the driving factors outlined in the study—such as job changes indicative of instability (career-driven), life events that may induce stress (non-work-specific-driven), or public professional updates hinting at dissatisfaction (cognitive-driven). Based on these insights, they should then develop targeted outreach interventions to re-engage potential boomerang candidates in a timely and personalized manner.

Third, Boomerang intention (cognitive commitment and behavioral enactment) positively influence the formation of the boomerang behavior. Building on the insight that boomerang intention—comprising cognitive commitment and behavioral enactment—directly drives the formation of boomerang behavior, organizations seeking to effectively attract former employees must move beyond passive relationship maintenance and proactively establish a systematic dual-path approach of “cognitive attraction and action enablement.” Specifically, companies should strengthen cognitive commitment by reframing “returning” as a desirable career advancement strategy—for instance, by integrating the concept of return into employer branding narratives and actively disseminating successful boomerang cases—thereby enhancing former employees’ perception of the value and feasibility of a comeback; simultaneously, they must lower the practical barriers to return by institutionalizing clear re-entry channels, such as dedicated alumni talent pools and streamlined rehiring processes with accelerated interviews and transparent policies, to transform intention into action.

Finally, organizational opportunity —encompassing talent gaps, value assessments, and industry ecology—determines the feasibility of boomerang returns. Organizations should therefore tailor their boomerang employment strategies based on their needs, industry characteristics, and talent value assessments. First, in industries with tight industry ecology, such as consulting or internet sectors, where collaboration and co-evolution are essential, implementing boomerang strategies can enhance industry connections, foster resource sharing, and promote complementary advantages. Therefore, companies operating within such industry ecosystems should place greater emphasis on boomerang employment. Second, for companies facing talent shortages, boomerang employment offers an efficient solution for talent replenishment, helping to maintain team stability and continuity. Therefore, organizations experiencing talent scarcity should proactively leverage boomerang recruitment as a strategic response. Moreover, given that higher-value talents are more likely to have the opptunity to return, organizations should develop detailed profiles and classifications of former employees. Under resource constraints, the Pareto Principle can guide efforts—prioritizing relationship maintenance and management for high-value alumni. At the same time, firms must strike a balance and avoid indiscriminate rehiring.

### 5.4 Limitations and future research directions

This study utilized both online secondary data and in-depth interviews to enhance the rigor of data collection. However, considering that boomerang behavior is constrained by the previous organization’s decision-making, future research should adopt triangulation methods, incorporating data from multiple sources such as former employees, their former supervisors, and colleagues. This multi-source approach would further improve data reliability and reduce bias from single data sources.

Second, boomerang behavior may vary significantly across different industries, organizational types, and cultural contexts. Future studies should explore both commonalities and contextual differences in the mechanisms underlying return behavior in specific settings. This will enable more targeted guidance for managing boomerang behavior in diverse cultural environments, industries, or organizational structures.

Lastly, while this study proposes a theoretical model of the mechanisms that generate boomerang behavior, it does not empirically test the constructs or their relationships. Future research should undertake empirical validation through large-scale surveys and quantitative methods to progressively examine and refine the proposed model.

## Data Availability

The raw data supporting the conclusions of this article will be made available by the authors, without undue reservation.
